# Costs of implementing community-based intervention for HIV testing in sub-Saharan Africa: a systematic review

**DOI:** 10.1186/s43058-021-00177-y

**Published:** 2021-07-05

**Authors:** Florida Uzoaru, Ucheoma Nwaozuru, Jason J. Ong, Felix Obi, Chisom Obiezu-Umeh, Joseph D. Tucker, Thembekile Shato, Stacey L. Mason, Victoria Carter, Sunita Manu, Rhonda BeLue, Oliver Ezechi, Juliet Iwelunmor

**Affiliations:** 1 College of Public Health and Social Justice, Saint Louis University, St Louis, MO USA; 2 Department of Clinical Research and Development, London School of Hygiene and Tropical Medicine, United Kingdom Central Clinical School, Monash University, Melbourne, Australia; 3 Health Policy Research Group, University of Nigeria, Nsukka, Nigeria; 4 Division of Infectious Diseases, School of Medicine, University of North Carolina at Chapel Hill, Chapel Hill, USA; 5 Clinical Sciences Department, Nigerian Institute of Medical Research, Lagos, Nigeria

**Keywords:** Economic evaluation, Implementation cost, HIV testing

## Abstract

**Background:**

Community-based interventions (CBIs) are interventions aimed at improving the well-being of people in a community. CBIs for HIV testing seek to increase the availability of testing services to populations that have been identified as at high risk by reaching them in homes, schools, or community centers. However, evidence for a detailed cost analysis of these community-based interventions in sub-Saharan Africa (SSA) is limited. We conducted a systematic review of the cost analysis of HIV testing interventions in SSA.

**Methods:**

Keyword search was conducted on SCOPUS, CINAHL, MEDLINE, PsycINFO, Web of Science, and Global Health databases. Three categories of key terms used were cost (implementation cost OR cost-effectiveness OR cost analysis OR cost-benefit OR marginal cost), intervention (HIV testing), and region (sub-Saharan Africa OR sub-Saharan Africa OR SSA). CBI studies were included if they primarily focused on HIV testing, was implemented in SSA, and used micro-costing or ingredients approach.

**Results:**

We identified 1533 citations. After screening, ten studies were included in the review: five from East Africa and five from Southern Africa. Two studies conducted cost-effectiveness analysis, and one study was a cost-utility analysis. The remainder seven studies were cost analyses. Four intervention types were identified: HIV self-testing (HIVST), home-based, mobile, and Provider Initiated Testing and Counseling. Commonly costed resources included personnel (*n* = 9), materials and equipment (*n* = 6), and training (*n* = 5). Cost outcomes reported included total intervention cost (*n* = 9), cost per HIV test (*n* = 9), cost per diagnosis (*n* = 5), and cost per linkage to care (*n* = 3). Overall, interventions were implemented at a higher cost than controls, with the largest cost difference with HIVST compared to facility-based testing.

**Conclusion:**

To better inform policy, there is an urgent need to evaluate the costs associated with implementing CBIs in SSA. It is important for cost reports to be detailed, uniform, and informed by economic evaluation guidelines. This approach minimizes biases that may lead decision-makers to underestimate the resources required to scale up, sustain, or reproduce successful interventions in other settings. In an evolving field of implementation research, this review contributes to current resources on implementation cost studies.

**Supplementary Information:**

The online version contains supplementary material available at 10.1186/s43058-021-00177-y.

Contributions to the literature

- This study highlights an important gap in scientific evidence in the economic evaluations of human immunodeficiency virus (HIV) prevention programs, that is, the need to disaggregate the costs of the resources needed for different components of their program.
- Despite HIV prevention programs reporting the use of a micro-costing approach, many cost components of program implementation were inadequately reported.
- This study synthesizes the growing literature on economic evaluations of HIV prevention programs, and by so doing advocates for an increased use of economic guidance for better reporting of the cost information for implementing HIV prevention programs in SSA.

## Background

Community-based interventions (CBIs) are interventions that may combine different strategies across multiple settings and are aimed at improving the well-being of the target population in a community [1]. These different strategies may include education about HIV prevention, promotion of HIV awareness, counseling about risk-reducing behaviors, and promotion of HIV testing and counseling [2]. With HIV prevention, CBIs aims to increase access to medical care to a population that are identified as at risk of HIV infection, such as intravenous drug users, sex workers, men who have sex with men (MSM), or young people with multiple sexual partners [3–7]. They do so by reaching these individuals in homes, schools, or community centers [2]. For uninfected individuals in Sub Saharan Africa (SSA), testing offers a critical point of contact with healthcare providers to use effective HIV prevention strategies; and for people living with HIV, testing provides a gateway to diagnosis and treatment [8]. However, implementing these interventions comes at a cost, and SSA nations will need to optimize their limited resources to scale up HIV prevention interventions that are high quality and cost-effective [9, 10].

Although understanding the costs associated with program implementation is critical to the adoption, success, and sustainability of the program [11, 12], little is known about the costs required to implement these community-based interventions [13]. Furthermore, itemized costs of the resources used to accomplish the different components of their program are infrequently reported by HIV studies implemented [14–18]. The implementation costs of interventions are contextual because the costs depend on the complexity of the intervention, the implementation strategy, and the intervention’s geographical and healthcare setting [12]. When cost analyses are reported in the form of “total cost,” as is common, without the breakdown of individual components of the total cost [18–21], they fail to provide crucial information on the individual factors driving the implementation costs [11, 22, 23]. Therefore, such cost studies may have limited application in implementation science as they are unable to present a realistic scenario of the programs’ implementation [24].

Micro-costing or an “ingredients” approach to costing provides a thorough understanding of the resources required for a project [23, 25]. These are a more transparent and precise approach to economic costing in healthcare because it involves identifying all resources used in an intervention [23, 25]. These costing approaches are recommended for studies focused on the implementation of HIV testing programs conducted in community-settings [25]. When HIV prevention program reports include detailed information about costs and outcomes, they present a realistic scenario of how these programs can be implemented in a real-world setting [17, 26–28]. Data from detailed cost evaluation reports are critical and relevant to policymakers and other stakeholder groups [11, 12]. Cost information for interventions also facilitates their adaptation in other settings [18, 28]. They also minimize biases that may lead to decision-makers underestimating the resources required to scale up, sustain, or reproduce successful interventions in other settings [29].

Two previous reviews have explored the implementation costs of HIV testing interventions in SSA: a 2002 systematic review by Creese et al. and a literature review by Hauck et al. [30, 31]. In both reviews, few of the included studies focused on HIV testing and none used micro-costing approach [30, 31]. As such, the reviews may have limited application since they did not present a realistic scenario of how these programs were implemented. To address this gap in the literature, this study presents evidence of the costs of implementation of HIV testing services in SSA, as well as how the costs of implementing these interventions were analyzed and reported.

## Methods

### Search strategy

We conducted a systematic review of English language publications that described the costs of community-based implementation of HIV testing, and reported our findings in accordance to the PRISMA checklist [32]. There was no date restriction for the publications. On 2 December 2019, and updated on 26 April 2020, keyword searches were performed on the following databases: SCOPUS, CINAHL, Web of Science, Global Health, PsycINFO, MEDLINE, and Google Scholar. Keyword selection for cost was guided by the taxonomy of implementation outcomes outlined by Proctor et al. [12]. The search strategy (see S1) was designed to capture studies that evaluated the implementation costs of behavioral interventions: randomized control trials and non-randomized control trials, pilot studies, or implementation of evidence-based interventions (EBIs) that have a quantitative economic element (i.e., costs and benefits). The EBIs are peer-reviewed programs with outcomes that are supported by rigorous empirical evidence of effectiveness [33]. The search terms did not include individual SSA countries. The reference lists of the systematic reviews [15–17, 30, 31, 34–79] were checked for relevant studies that may have not been identified by our search. See Table 1 for keyword search strategy.

**Table 1:** Search strategy

Search terms used in PubMed, modified and used in other databases	
Category	Search terms combined with AND
Cost related analysis	Implementation cost OR cost-effectiveness OR cost analysis OR cost-benefit OR marginal cost
Intervention type	HIV testing
Region	sub-saharan Africa OR sub saharan Africa OR SSA
Combined search	((implementation cost OR cost-effectiveness OR cost analysis OR cost-benefit OR marginal cost) AND (HIV testing)) AND (sub-saharan Africa OR sub saharan Africa OR SSA)
Search results:	
Database	Number of items
Pubmed	613
Ovid (APA PsycInfo, Journals@ovid full text)	553
Scopus:	27
Web of science	270
Global health	27
Cinahl	19
Google Scholar [search terms – allintitle: Africa AND HIV testing AND cost analysis + allintitle: Africa AND HIV testing AND cost-effectiveness)	10

### Screening strategy

The completed search results were downloaded into Endnote X9 for citation management, deduplication, and literature screening. Study titles and abstracts were initially screened by two independent reviewers using the following inclusion and exclusion criteria. Publications were excluded if they were systematic or scoping reviews, meta-analyses, briefing, debates and commentaries, study protocols, guidelines, meeting reports, conference abstracts, and poster presentations. Interventions related to pediatric HIV prevention or implemented outside of SSA, not HIV-related or not primarily focused on HIV prevention were similarly excluded. Also excluded were interventions designed for people living with HIV/AIDS (PLWHA), pharmaceutical interventions, or utilized HIV prevention strategies other than testing, i.e., treatment as prevention (TasP), universal test and treat (UTT), prevention of mother to child transmission (PMTCT), prevention programs for serodiscordant couples, and voluntary medical male circumcision (VMMC). Interventions utilizing mathematical or simulation models modeling for analysis were excluded as they do not fit the purpose of this study. Studies deemed not to have met the “detailed cost analyses” criteria for micro-costing or “ingredient approach,” in their methodology were excluded (i.e., non-identification of the cost of the individual components of the interventions’ resources). We included HIV testing studies that (1) were community-based intervention in SSA and (2) had intervention and control/comparison arm of the study; and (3) reported disaggregated cost data, i.e., broke down the components of the total cost into small items (e.g., per-diems, overhead or transport).

### Data extraction

Two reviewers independently extracted data from each selected study (FU and UN). A third reviewer (CO) conducted an independent crosscheck to identify and resolve any disagreements. We extracted data on intervention description, geographical setting, HIV prevalence, population, sample size, time horizon, perspective, sensitivity analysis, cost measurement used, discount rate, costing instrument or toolkit used (where applicable), and data collection type. We categorized studies by the testing strategy to compare intervention-specific results. The primary cost measurements of interest were total implementation cost and cost per unit of interest (e.g., cost per client tested, cost per HIV diagnosis). Study outcomes not related to cost analyses were not reported in this review. Given that the interventions were too different to allow for pooling [80–82] and our aim was not to compare cost across the ten studies included in this review, we did not inflate the costs to a common year.

### Risk of bias

To systematically compare the interventions, we evaluated the rigor of each intervention using the risk of bias that was developed by the Evidence Project for behavioral interventions for HIV interventions in low- and middle-income countries [83]. The tool consists of eight items: cohort, control or comparison group, pre-post intervention data, random assignment of participants to the intervention, random assignment of participants to assessment, follow-up rate of 80% or more, comparison group equivalent on socio-demographics, and comparison group equivalent at baseline on outcome measures [84]. The risk of bias was independently rated by FU and UN using the guideline outlined by Kennedy et al. 2019 [84].

### Quality appraisal

One of the objectives of this review was to evaluate how the implementation costs of HIV testing interventions in SSA were analyzed and reported. We used two study quality appraisal frameworks: Quality of Health Economic Studies (QHES) standardized framework [85] (which assessed the quality of the cost analysis itself) and the Consolidated Health Economic Evaluation Reporting Standards (CHEERS) [86] (which assessed the reporting quality of the economic evaluation). The QHES and CHEERS frameworks are included in Appendices A and B respectively (in Additional file A1 and A2) [87, 88].

## Results

**Fig. 1:**
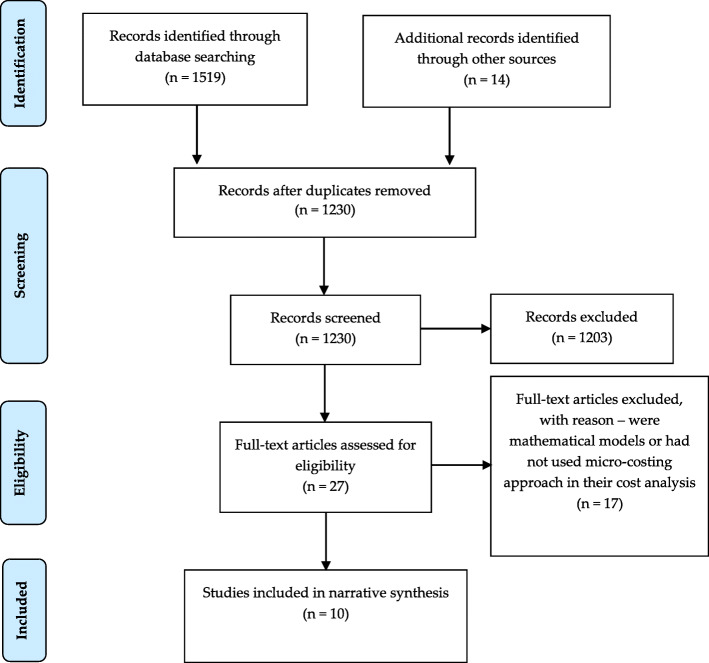
PRISMA table

We identified 1533 citations: 1519 from the database search, and 14 additional resources from previous studies on the cost of HIV interventions [30, 31]. Of 1533 articles, 25 were identified for full-text review. Seventeen of the 27 papers were excluded for not meeting the inclusion criteria. Seven provided total cost or cost per intervention outcome but did not have sufficient disaggregated costing data available [19, 89–94]. Thus, these 17 studies were excluded, leaving the remaining ten publications that met the full inclusion criteria [95–104]. Other reasons for exclusion included full text was unavailable [105, 106], studies had no control or comparison group [21, 107, 108], the study was a Universal Test and Treat (UTT) intervention [109], and studies were not primarily focused on HIV testing [110–112]. Although Chang et al. presented disaggregated data, the collection of cost data started 6 months after the start of the intervention when the intervention was believed to have reached a stable operational state per the goal of the study to characterize stable program functioning [113]. As such, the cost information provided by the study would not have fully reflected the implementation costs of the intervention. Figure 1 shows the PRISMA flowchart, and Table 2 shows the PRISMA checklist.

**Table 2:** PRISMA checklist

Section/topic	#	Checklist item	Reported in section
Title			
Title	1	Identify the report as a systematic review, meta-analysis, or both.	Title section
Abstract			
Structured summary	2	Provide a structured summary including, as applicable: background; objectives; data sources; study eligibility criteria, participants, and interventions; study appraisal and synthesis methods; results; limitations; conclusions and implications of key findings; systematic review registration number.	Abstract (page 1)
Introduction			
Rationale	3	Describe the rationale for the review in the context of what is already known.	Introduction
Objectives	4	Provide an explicit statement of questions being addressed with reference to participants, interventions, comparisons, outcomes, and study design (PICOS).	Introduction
Methods			
Protocol and registration	5	Indicate if a review protocol exists, if and where it can be accessed (e.g., Web address), and, if available, provide registration information including registration number.	Systematic review not registered
Eligibility criteria	6	Specify study characteristics (e.g., PICOS, length of follow-up) and report characteristics (e.g., years considered, language, publication status) used as criteria for eligibility, giving rationale.	Methods
Information sources	7	Describe all information sources (e.g., databases with dates of coverage, contact with study authors to identify additional studies) in the search and date last searched.	Methods
Search	8	Present full electronic search strategy for at least one database, including any limits used, such that it could be repeated.	Methods and Supplemental Table S0
Study selection	9	State the process for selecting studies (i.e., screening, eligibility, included in systematic review, and, if applicable, included in the meta-analysis).	Methods
Data collection process	10	Describe method of data extraction from reports (e.g., piloted forms, independently, in duplicate) and any processes for obtaining and confirming data from investigators.	Methods
Data items	11	List and define all variables for which data were sought (e.g., PICOS, funding sources) and any assumptions and simplifications made.	Methods
Risk of bias in individual studies	12	Describe methods used for assessing risk of bias of individual studies (including specification of whether this was done at the study or outcome level), and how this information is to be used in any data synthesis.	Methods
Summary measures	13	State the principal summary measures (e.g., risk ratio, difference in means).	Methods
Synthesis of results	14	Describe the methods of handling data and combining results of studies, if done, including measures of consistency (e.g., I^2^) for each meta-analysis.	Methods
Risk of bias across studies	15	Specify any assessment of risk of bias that may affect the cumulative evidence (e.g., publication bias, selective reporting within studies).	Methods
Additional analyses	16	Describe methods of additional analyses (e.g., sensitivity or subgroup analyses, meta-regression), if done, indicating which were pre-specified.	Supplemental Appendix (CHEERS and QHES framework)
Results			
Study selection	17	Give numbers of studies screened, assessed for eligibility, and included in the review, with reasons for exclusions at each stage, ideally with a flow diagram.	Results and Supplemental Figure S0
Study characteristics	18	For each study, present characteristics for which data were extracted (e.g., study size, PICOS, follow-up period) and provide the citations.	Result and Supplemental Table S1
Risk of bias within studies	19	Present data on risk of bias of each study and, if available, any outcome level assessment (see item 12).	Result and Supplemental Table S2
Results of individual studies	20	For all outcomes considered (benefits or harms), present, for each study: (a) simple summary data for each intervention group (b) effect estimates and confidence intervals, ideally with a forest plot.	Supplemental Table S1
Synthesis of results	21	Present results of each meta-analysis done, including confidence intervals and measures of consistency.	Meta-analysis not done
Risk of bias across studies	22	Present results of any assessment of risk of bias across studies (see Item 15).	Discussion
Additional analysis	23	Give results of additional analyses, if done (e.g., sensitivity or subgroup analyses, meta-regression [see Item 16]).	Results
Discussion			
Summary of evidence	24	Summarize the main findings including the strength of evidence for each main outcome; consider their relevance to key groups (e.g., healthcare providers, users, and policy makers).	Discussion
Limitations	25	Discuss limitations at study and outcome level (e.g., risk of bias), and at review-level (e.g., incomplete retrieval of identified research, reporting bias).	Discussion
Conclusions	26	Provide a general interpretation of the results in the context of other evidence, and implications for future research.	Discussion
Funding			
Funding	27	Describe sources of funding for the systematic review and other support (e.g., supply of data); role of funders for the systematic review.	Funding statement

From: Moher D, Liberati A, Tetzlaff J, Altman DG, The PRISMA Group (2009). Preferred Reporting Items for Systematic Reviews and Meta-Analyses: The PRISMA Statement. PLoS Med 6(6): e1000097. doi:10.1371/journal.pmed1000097

For more information, visit: www.prisma-statement.org

### Risk of bias

A study must meet at least one of these three criteria (cohort, control, or comparison group, pre-post intervention data) to be included in the review. We calculated the inter-rater reliability for each tool item. All items are treated as dichotomous, whereby we collapsed “not applicable” and “not reported” responses with “no” to reflect an assessment of whether the study did or did not get credit for having achieved that item. We added up the number of items met to create a final summary score for each study and using the weighted kappa assessed inter-reliability between the raters. The total count of agreement was substantial (κ_w_ = 0.73). No study was excluded from the review due to concerns about biases. The summary of the risk of bias rating is presented in Table 3.

**Table 3:** Risk of bias assessment

Study	Cohort	Control or comparison group	Pre/post intervention data	Random assignment of participants to intervention	Random selection of participants for assessment	Follow-up rate of 80% or more	Comparison group equivalent on socio-demographics	Comparison group equivalent at baseline on outcome measures
HIV self-testing								
Choko et al. 2019 [97]	Yes	Yes	NA	Yes	Yes	NA	Yes	Yes
George et al. 2018 [98]	Yes	Yes	NA	Yes	Yes	NR	Yes	Yes
Maheswaran et al. 2016 [99]	Yes	Yes	NA	Yes	Yes	NA	Yes	Yes
Home-Based HIV counseling and testing								
Bogart et al. 2017 [95]	Yes	Yes	NA	No	No	Yes	Yes	Yes
Cham et al. 2019 [96]	Yes	Yes	NA	No	No	NA	Yes	Yes
Mulogo et al. 2013 [101]	Yes	Yes	NR	No	No	NA	Yes	Yes
Tabana et al. 2015 [104]	Yes	Yes	Yes	Yes	Yes	NA	Yes	Yes
Mobile HIV counseling and testing								
Meehan et al. 2017 [100]	Yes	Yes	NA	NA	NA	NR	Yes	Yes
Parker et al. 2015 [103]	Yes	Yes	NA	NA	NA	No	Yes	Yes
Provider-initiated counseling and testing								
Obure et al. 2012 [102]	Yes	Yes	NA	NA	NA	NA	Yes	Yes

*NR* not reported, *NA* not applicable

### Characteristics of studies

The ten studies included in the review provided an economic evaluation of HIV testing interventions in SSA, either as a component of a larger study design [95, 96, 103] or as a stand-alone cost analysis [97–102, 104]. Four studies collected cost data retrospectively [96, 100, 102, 104]. Data from five studies were collected prospectively [95, 97–99, 101]. One paper did not disclose their study’s data collection method [103]. The study participants in four studies were 18 years and older [95, 97, 99, 101]. In Tabana et al., participants had to be 14 and older; 13 and older in Cham et al. [96, 104]. Overall, the interventions spanned a period of nine years, 2008–2017.

#### Study settings

All ten interventions were implemented in East and Southern Africa. Two studies were conducted in Kenya [98, 102], and two in Swaziland [102, 103]. The studies by Parker et al. and part of Obure et al. were both conducted in Swaziland, a landlocked lower-middle-income country in Southern Africa, with a population of 1.2 million, but in different locations Swaziland [102, 103]. The Parker et al. study was carried out in the relatively rural Shiselweni region with an estimated population of 41,000 of people living with HIV and 15,000 person who are unaware of their HIV status [114, 115]. There was no specific mention of the locations Obure et al. was conducted, only that it was in 41 health facilities in Kenya and Swaziland that were chosen to represent urban and rural regions [116]. Aside from the information that the George et al. study was conducted in Kenya, no additional location-based information provided in the article [98]. The article mentioned that The North Star Alliance, the organization George et al. partnered with, provided health services to hard-to-reach populations across Africa and that in 2017, the organization operated 53 clinics located at major transit hubs in 13 countries in Southern and East Africa, including eight in Kenya. Services provided by The North Star Alliance included HIV self-testing (HIVST), screening, and treatment of infectious disease (e.g., STI, HIV, TB, malaria) diagnosis and treatment of mobility-related and other non-communicable diseases, health education and laboratory services [98].

Two studies were carried out in Malawi: Choko et al. and Maheswaran et al. [97, 99]. In Choko et al. (2019), the specific location of the study was omitted. However, the article specified that a total of 3137 pregnant women (in 71 clusters with approximately 20–30 women per cluster) were initially screened for the study—the 36 clusters in the first stage of trial, and then 35 clusters in the second stage 2. From 3137, 2349 were included in the final study [97]. The Maheswaran et al. was conducted in three high-density urban suburbs of Blantyre with an adult population of approximately 34,000 residents, 1200 adults of which made it into the study [99].

Two studies were set in South Africa, Meehan et al. and Tabana et al. [100, 104]. In Meehan et al., the study took place in the Cape Metro district, Western Cape Province [100]. The study was carried out in partnership with The Desmond Tutu TB Centre (DTTC) at Stellenbosch University, and five non-governmental organizations (NGOs) in five peri-urban communities in the district characterized by poverty, overcrowding, high unemployment rates, and high HIV prevalence [117]. Tabana et al. (2012) was a cross-sectional study conducted in KwaZulu-Natal province, a sub-district with a population of approximately 243,000 people, with the highest HIV prevalence rate in South Africa (17%) and where 70% of the households lived below the poverty line [104]. Only 16% of the adult population in province had reportedly ever had HIV testing [118].

Mulogo et al. and Bogart et al. are two study carried out in Uganda [95, 101]. The study by Mulogo et al. was conducted in two sites: Mbarara and Isingiro districts. The populations of Mbarara and Isingiro districts are estimated to be 418,300 and 385,500 respectively [119]. Facility-based VCT was offered the Mbarara study site (Kabingo sub-county) while home-based VCT was offered to Isingiro study site (Rugando sub-county) [101]. For the 2017 Bogart et al. study was conducted in Wakiso District; event-based HIV testing in Zzinga Island, while and home-based HIV testing in Kavenyanja Island. Zzinga Island was estimated to have about 700 households, while Kavenyanja Island has about 1100 households [95].

The Cham et al. study was the only study included in this review that was conducted in Tanzania [96]. In Bukoba Municipal Council (BMC), the capital of Kagera Region is located on the western shore of Lake Victoria, with its economy supported by fishing and agriculture. As such, BMC residents are primarily fishermen and associated populations that support the fishing industry, including sex workers [96]. Fifty-two percent of men and 68% of women in BMC have reportedly received an HIV test in the past 2 years [120, 121].

#### Study design

Table 4 summarizes the methodological design of the studies evaluated and provides a descriptive overview of interventions reported in the ten reviewed studies. Four categories of intervention types were identified: HIV self-testing (HIVST) [97–99], home-based testing and counseling [95, 96, 100, 101, 104], mobile-based testing and counseling [103], and provider-initiated testing (PITC) [102]. These interventions were commonly compared to facility-based testing [97–99, 101, 104], event-based testing [95], home-based testing [103], PITC [96], and voluntary testing [102]. Four studies were randomized control trials; three of which evaluated the cost of implementing HIVST interventions [97–99] and one evaluated home-based testing [104]. Mulogo et al. was a longitudinal study with a pre-post cross-sectional investigative phase [101]. The remaining five studies did not state the study design, but the description of the data collection process suggests a cross-sectional design [95, 96, 100, 102, 103], whereby Bogart et al., Meehan et al., Obure et al., and Parker et al. were comparison group study, while Cham et al. was a cohort study. All ten studies were appraised for their QHES score.

**Table 4:** Descriptive overview of interventions

Reference	Intervention description		Geographical setting	HIV prevalence	Population	Sample size	Time horizon	Data collection type	Perspective	Cost measurement	Sensitivity Analysis	Discount rate	Analysis Instrument	QHES Score
	Intervention	Control(s)												
HIVST														
Choko et al. 2019 [97]	1. Standard of care + clinic access to HIVST [ST]. 2. ST + $3 conditional fixed cash financial incentive [ST + $3]. 3. ST + fixed cash financial incentive [ST + $10]. 4. ST+ 10% chance of winning $30 [ST + lottery]. 5. ST + phone reminder to present at clinic [ST + reminder]	Standard of care [SOC]	Blantyre, Malawi	Not stated	Women attending an antenatal care (ANC} for the first time for their current pregnancy (regardless of trimester), 18 years and older, with a primary male partner not known to be on ART	2349 pregnant women	8 August 2016 and 30 June 2017	Prospective	Healthcare only	Costs per male partner who attended the clinic with a confirmed HIV test result	None		None	52
George et al, 2018 [98]	Short message service (SMS) promoting availability of HIVST, sent once a week for 3 weeks [HIVST].	1. Standard of care (SMS reminder sent once. [SOC] 2. Enhanced standard of case (SMS reminder sent once a week for 3 weeks [Enhanced SOC].	Kenya	Not stated	Male truckers (Trucker) and female sex workers (FSW)	2262 truckers and 2196 FSWs	Dec. 2016 to April 2017	Prospective	Societal	(1) Total cost of cohort. (2) Total cost of intervention. (3) Cost per client. (4) Cost per additional client.	Univariate (cost sensitivity)	3%	Not stated	85.5
Maheswaran et al., 2016 [99]	Intervention: HIVST	Control: Facility-based HIV testing and counseling (FBHTC) in the following sites, 1. Queen Elizabeth Central Hospital [QECH] 2. Ndirande health center [Ndirande] Chilomani health center [Chilomani].	Blantyre, Malawi	18% (adult)	Adult	1,200 adults	Feb. 2013 to April 2014	Prospective	Societal	(1) Total annual health provider cost. (2) Cost per participant tested. (3) Cost per HIV positive identified. (4) Cost per HIV positive individual assessed for ART eligibility. (5) Cost per HIV positive initiated onto ART. (6) quality-adjusted life-year (QALY)	EuroQol EQ-5D (impact of alternative approaches to estimating total societal costs and for valuing health-related quality of life [HRQoL])	None	Non-parametric bootstrap methods and Generalized linear models (GLM) for multivariate analyses of cost data (stewed cost data).	86
Home-Based HIV counseling and testing														
Bogart et al. 2017 [95]	Home-based HIV testing and counseling [HBHTC]	Event-based testing and counseling [EBHTC]	Lake Victoria, Uganda	Not stated	Adult 18 years and above. Children under 12 years old with consent. Infant 18 months and younger, with HIV+ mothers and mothers’ consent.	1363 individuals in 629 households (965 adults, 386 children; 13 missing ages)	May–July, 2015	Prospective	Healthcare only	(1) Total cost of each intervention (2) cost per test	None	None	Not stated	59
Cham et al. 2019 [96]	Home-based HIV testing and counseling [HBHTC]	1. Provider-initiated testing and counseling [PITC]. 2. Venue-based HIV testing and counseling [VBHTC].	Bukoba Municipal Council (BMC), Tanzania	9.1% for adults aged 18–49 years (BMC)	People aged 15 years and older	133,695 people (56 304 males, 77 391 females).	2014–2017	Retrospective	Healthcare only	Estimated incremental cost over 2.5 years: (1) Total intervention cost (2) Total cost of testing Strategies. (3) Cost per test. (4) Cost per HIV diagnosed.	None	None	Not stated	76.0
Mulogo et al. 2013 [101]	Home-based HIV testing and counseling [HBHTC].	Facility-based HIV testing and counseling [FBHTC]	Rugando and Kabingo sub-Counties of Uganda	FBVCT = 7% HBVCT = 9%	1 adult per household between ages 18-59. Total number of participants = 971; 294 males and 677 females.	418,300 (Rugando) and 385,500 (Kabingo)	Nov. 2007 to March 2008	Prospective	Healthcare only	(1) Total annual economic cost. (2) Average cost per client counseled and tested. (3) Average cost per client diagnosed HIV positive	Univariate (HIV prevalence likely to change over time)	5%	TreeAge Pro 2009	79.5
Tabana et al. 2015 [104]	Home-based HIV testing and counseling [HBHTC].	Facility-based HIV testing and counseling [FBHTC]	KwaZulu Natal, South Africa	17%	Adults 18 years and above, individuals 14–17-year-old with guardian/parental consent)	100 to 200 households, approx. 46,000 people	Jan. to Dec. 2010	Retrospective	Healthcare only	(1) Total annual cost. (2) Cost per client tested. (3) Incremental cost per additional HIV test	Univariate (professional nurses’ salaries, catchment population size, HIV test kits)	3– 6%	CostIt software 2007	78.5
Meehan et al. 2017 [100]	Mobile-based HIV testing and counseling (MHTC)	Stand-alone community-based HIV testing and counseling [SAHTC].	Cape Metro district, Western Cape Province, South Africa.	Antenatal HIV prevalence of 20.4%	Not stated	5031 individuals (SAHTC) and 3104 (MHTC)	July to Sept. 2014	Retrospective	Healthcare only	(1) Total cost of each HTS modality. (2) Costs per persons counseled. (3) Costs per test. (4) Costs per diagnosis. (5)K Costs per HIV referred. (6) Costs per linked to HIV care.	None	None	Not stated	67.0
Mobile HIV counseling and testing														
Parker et al. 2015 [103]	Mobile-based testing and counseling (MHTC)	Home-based HIV testing and counseling [HBHTC].	Shiselweni region, Swaziland	Not stated	Individuals over 12 years of age who gave informed consent and deemed competent to make this decision. Individuals under 12, or lacking competence with legal guardian consent.	25 health facilities. 2043 (MHTC) and 12269 (HBHTC).	March - October, 2013	Not stated	Healthcare only	(1) Cost per person reached. (2) Cost per HIV positive identified. (3) Cost per HIV positive identified and linked to care.	None	None	Not stated	64.0
Provider-initiated counseling and testing														
Obure et al. 2012 [102]	Provider-initiated counseling and testing [PITC]	Voluntary counseling and testing [VCT].	Kenya and Swaziland	7.1% -Kenya 26%-Swaziland	Not stated	41 health facilities in Kenya and Swaziland	2008–2009 fiscal year	Retrospective	Healthcare only	(1) Total annual cost. (2) Average cost per client counseled and tested. (3) Average cost per client diagnosed HIV positive	None	3%	Not stated	45.5

#### Types of interventions

The only diagnostic testing reported in eight studies was HIV [95–101, 103]. In addition to HIV, participants in Tabana et al. were also tested for syphilis, gonorrhea, chlamydia, trichomonas, and candidiasis [104]. Furthermore, Tabana and colleagues did not disaggregate the costs for HIV testing specifically in the cost of the intervention. Although this is a study limitation, it was reported in the paper. In Obure et al., participants in the PITC arm of the study received routine healthcare (e.g., general primary care, maternal and child healthcare, care for sexually transmitted infections, and inpatient services) [102]. In Bogart et al., Meehan et al., and Tabana et al., condoms were given to participants [95, 100, 104]. Participants in Bogart et al. also received de-worming tablets, bed nets, and water guard tablets [95]. Seven studies stated the cadre of healthcare workers involved in the intervention [95, 96, 99–102, 104]. Nurses were used in five studies [96, 100–102, 104]; lay counselors in seven studies [95, 96, 99–102, 104]; and lab assistant/technologist in two studies [101, 102]. In three studies, lay counselors served both as pre- and post-test counselors as well as tested the participants [101, 102, 104].

### Types of costing measures

Costs were predominantly evaluated using a healthcare perspective (*n* = 8) [95–97, 100–104]. All but one study used empirical analytic approach [95–100, 102–104], with the exception using a model-based approach [101]. While Mulogo et al. mentioned the use of a decision model in their economic evaluation, the particular model used was not stated [101]. None of the studies mentioned the use of any economic evaluation guidelines to inform their costing approach. Tabana et al. was the only study that specified the costing instrument used in their study [104].

Confirmatory testing in a healthcare facility was required in four studies [96, 97, 99, 103]. In Meehan et al., HIV-positive clients were given referral letters to a public health facility for care and treatment [100]. However, none of these five studies reported who bore the cost of confirmatory testing or HIV treatment for participants who tested positive to HIV [96, 97, 99, 100, 102]. Though Maheswaran et al.’s study was a societal perspective, the authors did not state if an amount of money was paid out of pocket by the patient or was subsidized or paid for by the government or donor as part of the intervention. The cost of test kits was included in the intervention costs in seven studies [95–101], with Bogart et al., Choko et al., George et al., and Maheswaran et al. providing the individual cost of the kits [95, 97–99]. Maheswaran et al. reported the unit cost of purchasing and shipping the HIVST kits, as well as the cost of the finger-prick rapid diagnostic test (RDT) kits used in the health facilities [99]. Obure et al. and Parker et al. did not state if kits were free or subsidized or if it was purchased for the intervention [102, 103]. Tabana et al. costed testing equipment, without specifying the particular testing equipment [104].

Although all ten studies reported using micro-costing or ingredient-approach in their cost evaluation, individual costs of different implementation components were aggregated in many studies. In Meehan et al., the cost of all equipment and assets was aggregated as capital goods, while the cost of utilities, consumables, and services directly related to testing service was aggregated as recurring goods [100]. While Maheswaran et al. provided the most detailed cost information compared to other studies, capital/overhead was costed without the study stating what constituted capital/overhead in the program [99]. Notwithstanding, we identified 12 common resource types: start-up cost, material, and equipment, vehicle, fueling, stationary/supplies, office rental/building, utilities, furniture, maintenance, training, and transportation. Personnel cost, material/equipment, stationary/supplies, and training were the cost items typically presented as stand-alone cost components. Personnel costs were reported in all ten studies. Materials and equipment were reported in seven studies [95–99, 103, 104]. Stationary/supplies were reported in five studies [96–98, 101, 104], and training in four studies [96, 98, 99, 101]. Fueling or vehicle [96, 99, 100, 102], furniture or maintenance [96–100, 103, 104], and office rent/building or utilities [97–100, 103, 104] were commonly aggregated. Tabana et al. was the only study to report on start-up costs. Tabana et al., Maheswaran et al., and Mulogo et al. had the most detailed cost information [99, 101, 104]. Conversely, Obure et al. and Meehan et al. had the least [100, 102].

### Cost analysis

Six studies reported only the financial cost of implementing the interventions, focusing on the direct cost of the intervention [95–97, 101, 103, 104]. Four studies performed economic costing [98–100, 102]. In George et al., costs not specifically borne by the counseling and testing services were said to have been calculated but was not reported in the paper [98]. While Meehan et al. said economic costing was performed, the costs of free products were not accounted for in the paper [100]. Maheswaran et al. was the only study that reported cost for patient time-off, patient direct non-medical cost, and caregiver time [99]. Two studies stated they were conducting a cost-effectiveness analysis (CEA) [101, 104]. We identified Maheswaran et al. as a cost-utility analysis (CUA) because the study had measured the health-related quality of life (HRQoL) of the participants [99]. The remainder seven studies were identified as cost-effectiveness studies since they reported cost per unit outcome of interest [95–98, 100, 102, 103]. Eight studies reported the total cost per intervention [95–101, 104]. All ten studies reported the cost per unit of interest: cost per test [95, 96, 98–102, 104], cost per HIV diagnosed [96, 99–103], and cost per client linked to care [97, 99, 100, 103]. Table 5 contains detailed information about the cost outcomes of the interventions.

**Table 5:** Intervention cost per outcome

Publication	Continuum of care costed	Total implementation cost		Cost per client	
		Intervention	Control	Intervention	Control
HIVST					
Choko et al. 2019 [97] Intervention: HIVST^a^ Control: SOC	Testing and initiated ART treatment	Total intervention cost (excluding ART or VMMC): 1. ST^b^ = USD 3446.03 2. ST + $3 = USD 3678.44 3. ST + $10 = USD 7469.87 4. ST + lottery = USD 1175.64 5. ST + phone = USD 3 464.41	SOC = USD 557.40	1. Cost per male partner tested + attended male friendly clinics (MFC): a. ST = USD 40.54 b. ST + $3 = USD 23.73 c. ST + $10 = USD 28.08 d. ST + lottery = USD 39.19 e. ST + phone = USD 41.24 2. Cost per male partner tested + ART^c^/VMMC^d^: a. ST = USD 127.63 b. ST + $3 = USD 94.32 c. ST + $10 = USD 109.85 d. ST + lottery = USD 167.95 e. ST + phone = USD 157.47	1. Cost per male partner tested + attended MFC^e^: SOC^f^ = USD 9.95 2. Cost per male partner tested + ART/VMMC: SOC = USD 39.81
George et al. 2018 [98] Intervention: HIVST Controls: SOC and Enhanced SOC	Testing	Total intervention cost • Trucker = USD 3678.44 • FSW^g^ = USD 7469.87 1. Total cost per intervention (Trucker): a. HIVST = USD 544.03 2. Total cost per intervention (FSW): • HIVST = USD 925.55	1. Total intervention cost • SOC = USD 284.81 • Enhanced SOC = USD 335.69 2. Total cost per intervention (FSW): • SOC = USD 411.18 • Enhanced SOC = USD 473.04	1. Cost per client (Trucker): • HIVST = USD 20.92 2. Cost per client (FSW): • HIVST = USD 11.43 3. Cost per additional client tested (Trucker): • HIVST = USD 21.48 4. Cost per additional client tested (FSW): • HIVST = USD 15.80	1. Cost per client (Trucker): • SOC = USD 28.48 • Enhanced SOC = USD 33.57 2. Cost per client (FSW): • SOC = USD 9.56 • Enhanced SOC = USD 10.28 3. Cost per additional client tested (Trucker): • SOC = USD 26.26 • Enhanced SOC = USD 30.80 4. Cost per additional client tested (FSW): • SOC = USD 9.90 • Enhanced SOC = USD 10.63
Maheswaran et al. 2016 [99] Intervention: HIVST Control: FBHTC^h^	Testing, HIV diagnosis, and ART initiation	Total annual health provider cost: HIVST = USD 133 300	• Ndirande = USD 50 899 • Chilomani = USD 56 760 • QECH^l^ = USD 84 436	1. Direct cost per individual tested: HIVST = USD 8.78 2. Direct cost per HIV positive identified: HIVST = USD 97.50 3. Direct cost per HIV positive individuals assessed for ART eligibility: HIVT Service = USD 165.14 4. Direct cost per HIV positive individuals initiated onto ART: • HIVST = USD 319.67	1. Direct cost per individual tested: • Ndirande = USD 7.53 • Chilomani = USD 10.57 • QECH = USD 8.90 2. Direct cost per HIV positive identified: • Ndirande = USD 67.33 • Chilomani = USD 76.39 • QECH = USD 28.30 3. Direct cost per HIV positive individuals assessed for ART eligibility: • Ndirande = USD 83.48 • Chilomani = USD 92.38 • QECH = USD 37.73 4. Direct cost per HIV positive individuals initiated onto ART: • Ndirande = USD 109.85 • Chilomani = USD 132.42 QECH = USD 85.75
Home-based HIV counseling and testing					
Bogart et al. 2017 [95] Intervention: HBHTC^j^ Control: EBHTC^k^	Testing	HBHTC = USD 62 247	EBHTC = USD 25 780	HBHTC = USD 45.09	EBHTC = USD 46.99
Cham et al. 2019^1^ [96] Intervention: HBHTC Controls: PITC^l^ and VBHTC^m^	Testing and HIV diagnosis	1. Total intervention cost = USD 720 607.67 2. Total cost per intervention: HBHTC = USD 176 865.66	1. Cost per intervention • PITC = USD 404 364.89 • VBHTC = USD 139 377.12	1. Cost per test: HBHTC = USD 6.45 2. Cost per new HIV diagnosed: HBHTC = USD 354.44	1. Cost per test: • PITC = USD 4.55 • VBHTC = USD 7.98 2. Cost per new HIV diagnosed: • PITC = USD 123.66 • VBHTC = USD 372.67
Mulogo et al., 2013^2^ [101] Intervention: HBHTC Control: FBHTC	Testing and HIV diagnosis.	USD 3114	USD 2462	1. Cost per client tested: USD 5.0 2. Average cost per client diagnosed HIV positive: USD 1	1. Cost per client tested: USD 6.4 2. Average cost per client diagnosed HIV positive: USD 2
Tabana et al. 2015^3^ [104] Intervention: HBHTC Control: FBHTC	Testing	USD 233 239.02	USD 146 615.12	USD 29	USD 38
Mobile HIV Counseling and Testing					
Meehan et al. 2017 [100] Intervention: MHTC^n^ Control: SAHTS^o^	Testing, HIV diagnosis, referral to treatment.	USD 77 764	USD 96 616	1. Cost per person counseled: USD 25 2. Cost per person test: USD 25 3. Cost per HIV diagnosed: USD 1051 4. Cost per HIV referred: USD 1065 5. Cost per linked to HIV care: USD 2102	1. Cost per person counseled: USD 50 2. Cost per person test: USD 51 3. Cost per HIV diagnosed: USD 755 4. Cost per HIV referred: USD 773 5. Cost per linked to HIV care: USD 1039
Parker et al. 2015 [103] Intervention: MHTC Control: HBHTC	Testing and HIV diagnosis.			1. Cost per person reached: USD 24 2. Cost per HIV positive identified: USD 543 3. Cost per HIV positive identified and linked to care: USD 1698	4. Cost per person reached: USD 11 5. Cost per HIV positive identified: USD 343 6. Cost per HIV positive identified and linked to care: USD 797
Provider-initiated counseling and testing					
Obure et al. 2012 [102] Intervention: PITC Control: VCT^p^	Testing and HIV diagnosis.	Average annual economic cost 1. Kenya: USD 3 721 2. Swaziland: USD 10 407	Average annual economic cost 1. Kenya: USD 11 969 2. Swaziland: USD 16 716	1. Average cost per client: • Kenya: USD 5.71 • Swaziland: USD 7.79 2. Average cost per client diagnosed HIV positive: • Kenya: USD 46.96 • Swaziland: USD 47.85	1. Average cost per client: • Kenya: USD 8.27 • Swaziland: USD 9.44 2. Average cost per client diagnosed HIV positive: • Kenya: USD 110.32 • Swaziland: USD 45.56

Estimated incremental cost over 2.5 years;

^2^ICER = 3.50

^3^ICER = 19

^a^HIVST: HIV self-testing

^b^*ST* standard of care + access to HIVST

^c^*ART* antiretroviral therapy

^d^*VMMC* voluntary medical male circumcision

^e^*MFC* male friendly clinics

^f^*SOC* standard of care

^g^*FSW* female sex workers

^h^*FBHTC* facility-based HIV testing and counseling

^i^*QECH* Queen Elizabeth Central Hospital

^j^*HBHTC* home-based HIV testing and counseling

^k^*EBHTC* event-based HIV testing and counseling

^l^*PITC* provider-initiated testing and counseling

^m^*VBHTC* venue-based HIV testing and counseling

^n^*MHTC* mobile-based HIV testing and counseling

^o^*SAHTS* stand-alone community-based HIV testing and counseling

^p^*VCT* voluntary counseling and testing

In comparing the total implementation costs of the interventions to the controls, the latter was recorded to cost less in most studies. While this pattern was noted in all four categories of testing interventions (HIVST, home-based testing [HBHTC], mobile testing [MHTC], and provider-initiated testing [PITC]), the margin was wider with HIVST interventions. For instance, in Choko et al., total intervention cost (excluding ART/VMMC) for the five intervention strategies ranged from USD 1176 to USD 7470 [97]. The corresponding control (standard of care) cost was USD 557; less than half of what was spent implementing the least costly strategy. The margin was the narrowest in George et al.; USD 544 compared to USD 285 and USD 336 of implementing a standard of care and enhanced standard of care respectively [98]. Only Meehan et al. and Cham et al. reported implementing the intervention at a lower cost than the control [96]. However, in Cham et al., the total cost for the intervention (USD 176,866) was lower only for the PITC arm (USD 404,365) and not the venue-based testing service [VBHTC] (USD 139,377) [96]. However, cost per test and cost per HIV diagnosis was lowest in PITC of the three arms of testing modalities; VBHTC cost the most of the three [96].

When assessing cost per outcome, the cost of implementing the interventions was lower than that of the control for some outcomes but higher for other outcomes. For instance, in George et al., cost per client tested was lower for truck drivers: USD 20.92 for HIVST to USD 28.48 for the standard of care and USD 33.57 for an enhanced standard of care [98]. However, for female sex workers, the intervention group cost USD 11.43 to the USD 9.56 for the standard of care. In Bogart et al., there was minimal difference in the cost per test between intervention and control: cost per test for home-based testing was USD45.09 and USD46.99 for event-based testing as its control [95]. In Obure et al., the intervention arm of the study (PITC) costed less than the control (voluntary counseling and testing) for both total cost and cost per the two outcomes measure (per client and HIV diagnosed) [102].

The heterogeneity in reporting how different components of the intervention were costed made implementation cost*s* across studies incomparable. For instance, the financial input in Bogart et al. was calculated as cost per capita [95], percentage of total cost in Parker et al. [103], and cost per client tested in George et al. [98]. Eight studies adjusted for inflation and the dollar exchange rate relative the currency used in implementing the interventions. Three studies adjusted for inflation [96, 99, 104]. Maheswaran et al. used World Bank data to adjust all costs to account for inflation and differences in purchasing power between countries [99]. Cham et al. inflated the costs to 2017 price levels using the annual Tanzania consumer price index (CPI) ratio for 2014, 2015, and 2016 [96]. For Tabana et al., the costs incurred prior to 2010 were adjusted by using the CPI ratio for 2010 as the base year [104]. Costs in six studies were collected in local currencies and converted to US dollars [95, 98, 100–102, 104]; five of them provided the exchange rate used in converting to dollars [98, 100–102, 104]. Cham et al. and Tabana et al. annuitized the cost of some items [96, 104]: Cham et al. annuitized vehicle costs at an annual rate of 3% [96], while Tabana et al. annualized the economic costs of capital items, using either the items’ purchase value or replacement value, to an interest rate of 9% [104]. Only George et al. and Meehan reported the marginal costs alongside the absolute intervention costs, providing mainly the cost of additional test kits [98, 100]. In George et al., the cost of HIVST kit dropped from USD 9.22 to USD 2.00 after the agreement with Gates Foundation [98].

### Data quality appraisal

#### Quality of Health Economic Studies

Using the Quality of Health Economic Studies (QHES) checklist, 50% of ten studies were of high quality [96, 98, 99, 101, 104]. With a QHES score of 86%, Maheswaran et al. was the study with the highest quality [99]. At 46%, Obure et al. scored the lowest and was the only study of poor quality [102]. The QHES dimensions with the highest scores were questions responding to (a) if the study stated and justified the main assumptions and limitation of the study (90%); (b) if the presentation of study methods and analysis was clear and transparent (90%); (c) if the data extraction methodology was stated (90%); and (d) if the study conclusions/recommendations were justified and based on the study results (100%). Although economic evaluations are susceptible to, six studies failed to address how the researchers handled uncertainties [95–97, 100, 102, 103]. In that, the studies did not report performing statistical analysis to address random events or sensitivity analysis to cover a range of assumptions [95–98, 102, 103]. Three studies performed univariate sensitivity analysis [98, 101, 104]. Maheswaran et al. performed both sensitivity and statistical analyses for uncertainties [99]. For the seven studies with a time horizon beyond 1 year, four discounted for the effects and cost generated after the first year [98, 101, 102, 104]. Only George et al. reported CEA estimates from subgroup analyses: female sex workers and truck drivers [98]. Obure et al. was the only study that failed to disclose information of the data extraction method used [102]. Furthermore, the authors failed to state the perspective of their analysis and did not discuss the direction or magnitude of the potential biases of the study.

#### Consolidated Health Economic Evaluation Reporting Standards

Overall, the reviewed studies performed poorer on the Consolidated Health Economic Evaluation Reporting Standards (CHEERS) assessment compared to QHES. No study reached the 75% threshold to be classified as high quality. Four studies had scored lower than 50% and were therefore considered to be of poor quality [95, 97, 102, 103]. These studies also had the lowest QHES scores. Six studies were categorized as average quality, fulfilling between 50 and 63% of the criteria [96, 98–101, 104]. At 63% of criteria met, Maheswaran et al. had the highest CHEERS score [99]. Model choice and model assumptions were only applicable to Mulogo et al. [101]. However, the authors did not describe the assumptions underpinning the decision-analytic model and did not provide a figure showing the model structure as strongly recommended by CHEERS. Nine studies stated the time horizon for the costs being evaluated, but none justified why the time horizon was appropriate [95, 97–104]. Cham et al. did not state the study’s time horizon nor its appropriateness for the evaluation [96].

Six studies with time horizon more than a year failed to report the choice of the discount rate used and why it was appropriate [95–98, 100, 101, 103]. Nine studies did not characterize participants’ heterogeneity in their results [95, 96, 98, 100–104, 122]. Eight studies did not declare information about conflict of interest among study contributors [95–98, 101–104]. Seven items on the checklist were most commonly reported: a structured abstract [95–101, 104], explicit statement about the broader context of the study and its policy relevance in the introduction [95–97, 99–101, 103, 104], and a summary of population characteristics [95–100, 102, 103]. However, none of the seven studies that provided a structured abstract mentioned performing uncertainty analyses as required by CHEERS. The overall quality of the included studies according to the QHES and CHEERS checklists is summarized in Table 6.

**Table 6:** Overall data quality score

Publication	QHES^a^	CHEERS^b^
Bogart et al. 2017 [95]	Fair	Poor
Cham et al. 2019 [96]	High	Average
Choko et al. 2017 [122]	Fair	Poor
George et al. 2018 [98]	High	Average
Maheswaran et al. 2016 [99]	High	Average
Meehan et al. 2017 [100]	Fair	Average
Mulogo et al. 2013 [101]	High	Average
Obure et al. 2012 [102]	Poor	Poor
Parker et al. 2015 [103]	Fair	Poor
Tabana et al. 2015 [104]	High	Average

^a^QHES score category: ≥ 75 = high, 50–74 = fair, 25–49 = poor, 0–24 = extremely poor

^b^CHEERS score category: ≥ 75 = high, 50–74 = average, 0–49 = poor

## Discussion

We identified four categories of HIV testing interventions in this review: HIVST, home-based testing, mobile-based testing, and PITC. Three categories of testing services commonly served as controls: facility-based testing (FBHTC), event-based testing, and PITC. In two studies conducted in Malawi, the HIVST intervention costs twice as much the FBHTC, irrespective of the clinic site [97, 99]. Given that HIVST is a relatively new testing modality compared to the controls, the large difference in costs associated with implementation is partly attributable to the latter requiring little or no additional cost-intensive resources such as office rental, vehicle, or pre-implementation costs. Regardless, HIVST has the potential to increase uptake of HIV testing among undiagnosed people living with HIV and individuals with high HIV risk [123–126]. HIVST also provides complementary coverage to the standard HIV testing service [127]. With the release of the World Health Organization (WHO) guidelines to encourage HIVST [125, 128], our review findings make an important contribution to scaling up HIVST interventions in SSA.

Delivering PITC mostly costs the least whether as the intervention arm or control arm. This could be because PITC had been recommended by the WHO since 2007 [129]. Per recommendation, all patients attending health facilities are required to be routinely offered HIV testing in countries with generalized HIV epidemics [129]. Correspondingly, some costs associated with implementing PITC had already been built into the healthcare system. Nevertheless, while PITC may be low-cost, it is an approach with limited impact in reaching the greatest number of people [130–133]. Specifically, PITC does not reach individuals who do not typically utilize facility-based health services and other vulnerable or marginalized population groups with both high HIV incidence rates and low uptake of HIV testing due to fear of stigmatization (e.g., adolescents and men who have sex with men) [130–133]. Hence, the push for HIVST to address these barriers [123–126]. An example of how HIVST addressed some of the barriers associated with accessing clinic-based HIV testing services is in men’s health—their reluctance to visit healthcare facilities [118], thereby leading to a situation where there are a high proportion of HIV positive men who remain unaware of their HIV status [134]. HIVST is thought to offer an approach to improving men’s HIV testing rates by enabling the men convenience in time and place of conducting and interpreting their own HIV tests at their own convenient time and in a private space [135–137].

While all ten studies in the review presenting disaggregated cost information, the level of details varied across papers. Nevertheless, most studies provided fewer details about the individual cost of resources involved with accomplishing the different components of their program. Furthermore, there were many aspects of program implementation that were inadequately covered in the studies, such as startup costs related to preparatory work, and education or costs related to ongoing monitoring. Other than Tabana et al. [104], none of the other studies provided a clear picture of how much it cost to initiate the intervention or at what stage the cost analysis began. Nine out of the ten studies did not provide an explicit assessment of the “hidden” costs of implementation, such as an estimation of the cost of human or material resources that may have been free to the intervention or costs shared. Furthermore, the marginal costs of the interventions were reported by only two studies. While absolute costs are important for implementation planning as it presents the resource demands of an intervention design [138], marginal costs should not be neglected. This is because marginal costs capture how additional costs change as service levels increase, thereby making the reported cost information amenable for analysis and comparison [139, 140].

The studies did not provide details on whether the funds used in their programs were from one source or from multiple sources. As it pertains to personnel cost, it was also not clear if there had been a need to recruit new staff as the intervention advanced. If additional staff had been recruited, at what stage did it become necessary to do so and what extra cost was added to the intervention. This might be due to reporting bias or that they were only available in grey literature, thus limiting access to valuable information decision-makers need. As a result, the findings limit a realistic reflection of the resources that may be required to scale up, sustain, or reproduce the intervention in other settings. Subsequently, decision-makers may underestimate the cost of implementing the intervention and overestimate their benefits [18]. Detailed overview of the materials and personnel resources necessary for implementation facilitates budgeting, and enable implementers intending to adapt the intervention anticipate costs they may not otherwise consider [18]. This is a gap that needs to be filled by future researchers and program implementers.

Another critical gap to be addressed is the quality of the economic evaluations, particularly the reporting. Presumably, this is due to the low capacity of health technology assessment (which has economic evaluation at its core) in SSA [141]. Therefore, deliberate efforts will need to be made in other interventions/studies to build this capacity [141]. Most of the reviewed studies were generally of good quality using the QHES checklist, with half of them reaching the threshold for high quality (75%). Additionally, data were collected prospectively in half of the studies included in this review which minimized the risk of bias analyzing programs’ financial records retrospectively are subject to [18]. However, there still remains room for improvement. One way may be in calculating implementation costs in a manner consistent with existing guidelines such as Guideline for Economic Evaluations in Healthcare [142], Costing Guidelines for HIV/AIDS Intervention Strategies [143], or Reference Case for Estimating Cost of Global Health Services and Interventions [144]. These guidelines allow for more informative reports that aid decision-makers’ choices about the options available to them [145, 146]. Greater attention also needs to be paid to the reporting of the cost evaluation, as evidenced by the low quality of the CHEERS appraisal. This provides a premise for building capacity for economic evaluation in sustainable and institutionalized ways in SSA. Overall, implementation researchers should be mindful of the importance of reporting the cost of their implementing their interventions [147]. More so, they need to go beyond reporting cost-effectiveness and cost-benefit analyses to demonstrate the long-term economic effects of their interventions [148]. The absence of implementation cost data constrains deliberations about resources to consign to community-based health programs [149]. It constricts investment to program components like personnel, equipment, and modalities that are critical to strengthening and developing community health systems [149].

Although this review offers a synthesis of cost analyses of HIV testing intervention in SSA, there are potential limitations to this study worth mentioning. While the literature search was wide, the study had strict inclusion criteria, thus limiting generalizability [150, 151]. Nonetheless, the strictness of the criteria meant that the review was more concise, cohesive, and had fewer challenges potentially introduced by heterogeneity [152].

## Conclusions

Our systematic review shows that more attention needs to be paid to increasing the quality of conducting and reporting economic evaluations for HIV prevention interventions in SSA. Particularly, considerable effort needs to go into reporting them appropriately. To better inform policy, future evaluation of HIV prevention intervention will need to follow evidence-based guidelines and quality assurance frameworks so that the costs reported are extensive enough to address the many aspects of implementation that were not reported in previous evaluations. The interventions included in this review were disproportionately from East and Southern Africa. Geographic diversification of implementation cost analysis studies from West and Central Africa is needed in future research. As noted, implementation costs are contextual, thus costs of implementing HIV testing in West and Central Africa may or may not be substantially different compared to East and Southern Africa. Therefore, geographic diversification of implementation cost analysis studies from West and Central Africa to address the research question is needed. In an evolving field of implementation research, the review contributes to current resources on quantitative evaluation of cost studies. It particularly advocates for an increased use of economic evaluation guidance to aid implementation researchers for better reporting of cost information.

## Supplementary Information

**Additional file 1.** Appendix A: Quality of health economic studies framework.

**Additional file 2.** Appendix B: CHEERS checklist of items studies should include when reporting economic evaluation of health interventions.

**Additional file 3.** Quality of health economic studies framework.

## Data Availability

All data generated or analyzed during this study are included in this published article and its supplementary data.

## Abbreviations

AIDS: Acquired immunodeficiency syndrome
ANC: Antenatal care
ART: Antiretroviral therapy
CBI: Community-based intervention
CEA: Cost-effectiveness analysis
CHEERS: Consolidated Health Economic Evaluation Reporting Standards
CO: Chisom Obiezu-Umeh
CPI: Consumer price index
CUA: Cost-utility analysis
EBHTC: Event-based HIV testing and counseling
EBI: Evidence based intervention
EBP: Evidence-based practices
FBHTC: Facility-based HIV testing and counseling
FO: Felix Obi
FSW: Female sex workers
FU: Florida Uzoaru
GLM: Generalized linear models
HBHTC: Home-based HIV testing and counseling
HIV: Human immunodeficiency virus
HIVST: HIV self-testing
HRQoL: Health-related quality of life
JI: Juliet Iwelunmor
JJO: Jason J. Ong
JT: Joseph D. Tucker
MFC: Male friendly clinics
MHTC: Mobile-based HIV testing and counseling
OE: Oliver Ezechi
PITC: Provider-initiated testing and counseling
PLWHA: People living with HIV/AIDS
PMTCT: Prevention of mother to child transmission
QALY: Quality-adjusted life-year
QECH: Queen Elizabeth Central Hospital
QHES: Quality of Health Economic Studies
RB: Rhonda BeLue
RDT: Rapid diagnostic test
SAHTS: Stand-alone community-based HIV testing and counseling
SM: Sunita Manu
SLM: Stacey L. Mason
SMS: Short message service
SOC: Standard of care
SSA: Sub-Saharan Africa
ST: Standard of care + access to HIVST
TasP: Treatment as prevention
TS: Thembekile Shato
UN: Ucheoma Nwaozuru
UNAIDS: The Joint United Nations Programme on HIV and AIDS
USD: US Dollars
VBHTC: Venue-based HIV testing and counseling
VC: Victoria Carter
VCT: Voluntary counseling and testing
VMMC: Voluntary medical male circumcision
WHO: World Health Organization
