# Supplementary material for: Costs of implementing community-based intervention for HIV testing in sub-Saharan Africa: a systematic review
Source: Implement Sci Commun. 2021 Jul 5;2:73. doi: 10.1186/s43058-021-00177-y (PMC8259076; doi:10.1186/s43058-021-00177-y)
Supplement: Supplementary file 1 — Additional file 1. Appendix A: Quality of health economic studies framework. [file 43058_2021_177_MOESM1_ESM.docx]

| **Appendix A: Quality of health economic studies framework** | | |
| --- | --- | --- |
| **Number** | **Question text** | **Scoring** |
| 1 | Was the study objectively presented in a clear, specific and measurable manner? | Clear, specific, measurable = 7  Any two = 5  Any one = 2  None = 0 |
| 2 | Was the perspective of the analysis (societal, third party, payer, etc.) and reasons for its selection stated? | Perspective = 2  Reasons = 2  Both = 4 |
| 3 | Were variable estimates used in the analysis from the best available source (i.e. randomized control trial—best, expert opinion—worst)? | Randomized control trial = 8  Non-randomized control trial = 7  Cohort studies = 6  Case-control/case report/case series = 4  Expert opinion = 2 |
| 4 | If estimates came from a subgroup analysis, were the groups prespecified at the beginning of the study? | Yes = 1  No = 0 |
| 5 | Was uncertainty handled by (1) statistical analysis to address random events, (2) sensitivity analysis to cover a range of assumptions? | Statistical analysis = 4.5  Sensitivity analysis = 4.5  Both = 9 |
| 6 | Was incremental analysis performed between alternatives for resources and costs? | Yes = 6  No = 0  CCA type of economic evaluation = NA |
| 7 | Was the methodology for data extraction (including the value of health states and other benefits) stated? | Yes = 5  No = 0 |
| 8 | Did the analytic horizon allow time for all relevant and important outcomes? Were benefits and costs that went beyond 1 year discounted (3% to 5%) and justification given for the discount rate? | Time horizon = 3  Cost discounting = 1  Benefit discounting = 1  Justification = 2  All but justification = 5  All = 7 |
| 9 | Was the measurement of costs appropriate and the methodology for the estimation of quantities and unit costs clearly described? | Appropriateness of cost measurement = 4  Clear description of methodology for the estimation of quantities = 2  Clear description of methodology for the estimation of unit costs = 2  All = 8 |
| 10 | Were the primary outcome measure(s) for the economic evaluation clearly stated and did they include the major short-term? Was justification given for the measures/scales used? | Primary outcome clearly stated = 2  Include major short-term outcome = 2  Justification = 2  All = 6 |
| 11 | Were the health outcomes measures/scales valid and reliable? If previously tested valid and reliable measures were not available, was justification given for the measures/scales used? | Yes = 7  No = 0 |
| 12 | Were the economic model (including structure), study methods and analysis and the components of the numerator and denominator displayed in a clear, transparent manner? | Economic model = 2  Study methods = 1.5  Analysis = 1.5  Components of numerator = 1.5  Components of denominator = 1.5  All = 8  If not a modelling study, done for  Study methods = 2  Analysis = 2  Components of numerator = 2  Components of denominator = 2  All = 8 |
| 13 | Were the choice of economic model, main assumptions and limitations of the study stated and justified? | Economic model = 2  Assumptions = 2.5  Limitations = 2.5  All = 7  If not a modelling study, done (stated and justified) for  Assumptions = 3.5  Limitations = 3.5  Both = 7 |
| 14 | Did the author(s) explicitly discuss direction and magnitude of potential biases? | Direction = 3  Magnitude = 3  Both = 6 |
| 15 | Were the conclusions/recommendations of the study justified and based on the study results? | Yes = 8  No = 0 |
| 16 | Was there a statement disclosing the source of funding for the study? | Yes = 3  No = 0 |
